# Artificial Teeth

**Published:** 1881-01

**Authors:** 


Artificial Teeth manufactured by Mr. H. D. Justi, 516 Arch Street, Philadelphia, we find, in a long practice, are superior in natural beauty and strength. Many thanks, Mr. Justi, for those beautiful samples.

